# Deciphering the genetic control of innate and adaptive immune responses in pig: a combined genetic and genomic study

**DOI:** 10.1186/1753-6561-5-S4-S32

**Published:** 2011-06-03

**Authors:** Laurence Flori, Yu Gao, Isabelle P Oswald, François Lefevre, Marcel Bouffaud, Marie-José Mercat, Jean-Pierre Bidanel, Claire Rogel-Gaillard

**Affiliations:** 1 INRA, UMR de Génétique Animale et Biologie Intégrative, Jouy-en-Josas, France; 2 CEA, DSV, iRCM, Laboratoire de Radiobiologie et Etude du Génome, Jouy-en-Josas, France; 3 AgroParisTech, UMR de Génétique Animale et Biologie Intégrative, Jouy-en-Josas, France; 4 INRA, Laboratoire de Pharmacologie Toxicologie, Toulouse, France; 5 INRA, Laboratoire de Virologie et Immunologie Moléculaires, Jouy-en-Josas, France; 6 INRA, Station de contrôle de performances, UE450, Le Rheu, France; 7 IFIP, Pôle amélioration de l’animal, La Motte au Vicomte, France

## Abstract

Improving animal robustness and resistance to pathogens by adding health criteria in selection schemes is one of the challenging objectives of the next decade. In order to better understand the genetic control of immunity in French Large White pigs, we have launched a program combining genetic and genomic studies not focussing on any particular pathogen. Animals recorded for production traits were scored for a wide range of immunity parameters three weeks after vaccination against *Mycoplasma hyopneumoniae*: i) total white blood cells and lymphocyte counts and proportions of various leucocyte subsets including cells harbouring IgM, γδTCR, CD4/CD8, CD16/CD2 and CD16/CD172a/MHCII, ii) innate immune response parameters (phagocytosis and *in vitro* production of IL1B, IL6, IL8, TNF, IL12 and IFNαafter blood stimulation), iii) adaptive immune response parameters (lymphocyte proliferation, *in vitro* production of IL2, IL4, IL10 and IFNγ after blood stimulation, total IgG, IgA, IgM and specific IgG levels) and iv) two acute phase proteins (C-reactive protein and haploglobin). Across traits, heritability estimates reached 0.4 on average (se=0.1) and 42 of the 54 measured parameters showed moderate to high heritabilities (≥0.2), confirming that many parameters are under genetic control and could be included in selection protocols. Functional analyses revealed that the blood transcriptome is informative for part of the immunity traits and should provide relevant phenotypic information to better characterize some immunity traits.

## Background

In pig, genetic selection programs associated with the use of drastic sanitary rules and medical measures have proven their efficiency to improve production traits during the last 30 years. However, new concerns about European legislation, animal welfare and consumer safety, have recently forbidden the use of antibiotics for growth promotion and tended to decrease medical prophylaxis. In addition, emerging and chronic pathologies exist in pig farms that lead to major economic losses. In this context, improving robustness and resistance of animals to pathogens is a high priority in pig as in most livestock species. Underpinning health criteria to future animal breeding schemes that will select animals more globally resistant to various infectious diseases is one of the challenging objectives of the next decade, which would enable producers to meet new requests from legislations, consumer expectation and the pig economic sector.

Definition and use of multi-resistance criteria to infectious diseases are complex issues. Instead of working directly on resistance to specific pathogens, one alternative strategy consists in focusing on pig immunocompetence and to choose immune response (IR) parameters as health criteria [1,2]. For this purpose, it is necessary to identify IR parameters that are heritable and positively correlated with health or disease resistance. In addition, analysis of correlations between health traits and production traits is a complementary prerequisite before introducing such new parameters in existing selection schemes. Previous genetic studies have already reported medium to high heritabilities for several IR parameters such as total leukocyte counts and proportions of leukocyte subsets [3-6], delayed-type hypersensitivity reactions [2,7], proliferative response and cytokine (IL2 and IFNα) production after leukocyte stimulation [3], phagocytosis [3], total and specific antibodies [2-4,7] and acute phase proteins [4,5]. A divergent selection based on an index including four IR parameters has been followed up for eight generations [1,8] and quantitative trait loci (QTL) controlling total leukocyte count [3,9], mitogen-induced proliferation [3], IFNγ and IL10 production [10] and specific antibodies [3] have been reported. Taken together these results suggest that a selection based on IR parameters is feasible.

In order to provide complementary data and a global view of the genetic control of the whole innate and adaptive IR, we have launched a program aiming at characterizing immunocompetence of French Large White pigs after vaccination. The project does not target any specific pathogen and combines both genetics and functional genomics studies. The genetic approach includes i) measurement of numerous IR parameters covering innate and adaptive IR, ii) estimation of parameter heritabilities and iii) estimation of phenotypic and genetic correlations between these parameters and between these parameters and major production traits. The functional genomics approach is based on the analysis of the leukocyte transcriptome from peripheral blood using a DNA microarray specifically constructed to target immunity-related genes in pig [11]. Our first results on heritability and transcriptome analysis are presented in this report.

## Methods

### Animals, vaccination and blood sampling

A French Large White population (dam line) of 443 castrated male pigs raised in a test station was used in the study. Pigs were born in various breeding herds and transferred to the test station when 35 days-old with no prior vaccination. Animals considered in this survey arrived over an 18-months period. This population was subdivided into 307 nuclear families from 106 sires. All pigs were apparently healthy with no clinical sign of infection. They were recorded for various routinely measured production traits, which comprised growth and carcass measurements (19 parameters) as well as meat quality traits (nine parameters). All animals were vaccinated against *Mycoplasma hyopneumoniae* (Mh, Stellamune, Pfizer, one injection) one day after arrival in the test station. Three weeks after vaccination, blood was sampled via the external jugular vein from approximately 60 days-old animals and collected in EDTA-coated tubes, sodium heparin-coated tubes, PAXgene™ tubes (PreAnalytiX) and in tubes without any anti-coagulant. The experiment was conducted in accordance with the national regulations for humane care and use of animals in research.

### Immunity parameters

The list of IR parameters included in the study and their associated assays for phenotyping are described in Additional file 1 and protocols are detailed in Flori, unpublished. Briefly, hemogram parameters (total number of leucocytes, lymphocytes, monocytes, neutrophils, eosinophils, erythrocytes, hematocrit, red cell distribution width and platelets) were measured on blood using a MS4-5 counter (ElitechGroup, France). Leucocyte subsets characterized by membrane surface markers (IgM, TCRγδCD2/CD16, CD16/MHCII/CD172a and CD4/CD8) were quantified by Fluorescence-Activated Cell Sorting (FACS) using FACScan and CELLQuest software (Becton Dickinson, UK). Levels of non specific IgM, IgG, IgA and specific IgG directed against *Mycoplasma hyopneumoniae* were measured in pig serum by Enzyme-linked immunosorbent assay (ELISA) [12]. Haptoglobin and C reactive protein were measured in pig serum by colorimetric tests (Phase Haptoglobin Assay, ABCYS Biologie, France) and ELISA assays (Porcine C reactive Protein Assay, ABCYS Biologie, France), respectively. IL1B, IL6, IL8, TNF and IL12 were measured in the supernatant of blood stimulated for 24 hours with a mixture of lipopolysaccharide (LPS), Phorbol Myristate Acetate (PMA) and ionomycin using commercial ELISA kits (DuoSet ELISA development kits, R&D Systems, USA). IL2, IL4, IL10 and IFNγ were measured in the supernatant of blood stimulated with either LPS, or PMA-ionomycin or concanavalin A (CONA) for 48 hours using in-laboratory developed ELISA tests. IFNα activity was measured after stimulation by PK15 infected by the pseudorabies virus for 16 hours, using in-laboratory developed ELISA tests. Phagocytosis was measured on total blood with the Phagotest kit (ORPEGEN Pharma, Heidelberg, Germany). Lymphocyte proliferation was calculated after blood stimulation by either PMA-ionomycin, or LPS or CONA by measuring incorporation of ^3^H-methyl-thymidine (ICN, France) with a Liquid Scintillation Beta Counter (Kontron Instruments, France).

### Genetic analysis

Preliminary statistical analyses were performed using R software [13]. Q-Q plots and d’Agostino-Pearson omnibus tests were used to check the normality of parameter distribution. Parameters were normalized using a boxcox or ln (1+x) transformation. Significant effects of age at the time of vaccination, time of vaccination, herd of origin and time of experiment were detected with ANOVA, [13]. Variance components, genetic parameters and their standard errors were estimated using the REML methodology [14] applied to univariate mixed linear animal models with ASReml software [15], which included in the fixed part age at the time of vaccination, vaccination time, herd of origin and time of experiment effects, and in the random part a common litter environmental effect and direct genetic effects. For heritability estimations, a 95% confidence interval (95CI) was calculated.

### RNA extraction and transcriptome analysis

Total RNA was extracted from blood collected in PAXgene tubes using the PAXgene Blood RNA Kit (Qiagen, France). A porcine generic DNA chip enriched in immunity-related genes was used [11]. Five µg of total RNA from both blood samples and the reference sample (RNAs derived from a pool of tissues) were reverse-transcribed and directly labeled by Cy3 and Cy5, respectively, using the ChipShot^TM^ Direct Labeling System (Promega, USA). The CyDye-labeled cDNAs were purified by ChipShot^TM^ Membrane Clean-Up System (Promega, USA) and 750 ng each of Cy3-labeled and Cy5-labeled cDNA targets were combined for slide hybridization. Slides were scanned with an Agilent DNA Microarray scanner and array images processed with the GenePix^TM^ Pro software V6.0 (MDS Inc., Canada). Differentially expressed genes were established using statistical tests available in the version 2.12.0 of Limma (part of the Bioconductor package [13]).

## Results and discussion

### Immunity-related phenotypes

A total of 54 immunity-related parameters were measured that cover a wide range of innate and adaptive immune traits. Cells associated to innate IR include γδT lymphocytes, monocytes, natural killer cells, neutrophils and eosinophils and cells involved in adaptive IR include CD4^+^/CD8^+^, CD4^+^/CD8^-^, CD4^-^/CD8^+^ αβT lymphocytes and B lymphocytes. Innate immunity was also characterized by measuring phagocytosis efficiency, production of IL1B, IL6, IL8, TNF, IL12, IFNα in the supernatant of stimulated blood. In addition, serum levels of two inflammatory acute phase proteins were recorded (haptoglobin and CRP). For adaptive immunity, humoral IR was studied by measuring total antibodies (IgM, IgG and IgA) and specific IgG directed against *Mycoplasma hyopneumoniae* and cell-mediated IR were targeted by measuring Th1 (IL2 and IFNγ) and Th2 (IL4 and IL10) cytokine levels in stimulated blood supernatants and proliferation of mitogen-activated lymphocytes. Depending on parameters, 86 to 99 % of the animals have been recorded. According to d’Agostino-Pearson omnibus test (P-value = 0.05), none but CD16^-^/CD2^+^ cell parameter checked the normality assumption that led us to normalize all parameters as described in the Methods section. For most parameters (P-value=0.05), significant effects were found for age at the time of vaccination, time of vaccination, herd of origin and time of experiment.

### Heritabilities

**Figure 1:**
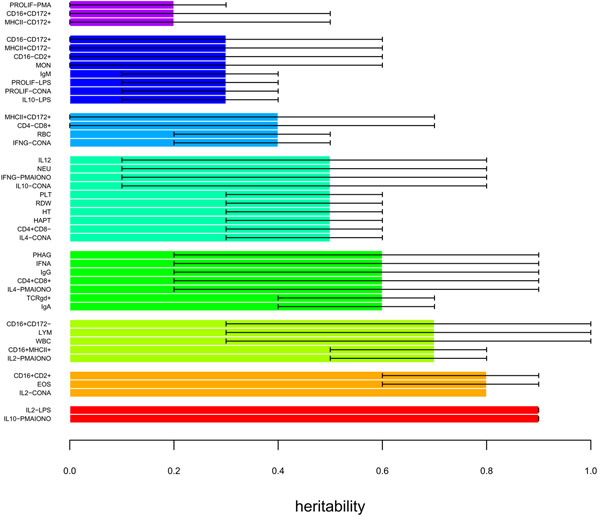
**Heritabilities of IR parameters.** Heritability estimations equal to or higher than 0.2 are shown with their associated 95CI for each parameter.

Genetic analyses showed that 42 parameters present moderate to high heritabilities (h^2^≥0.2; Figure 1). Interestingly, significant heritability estimates were obtained for parameters retrieved from various assays provided by cell counting or measured from serum or *in vitro* stimulated cells, suggesting that the range of heritable parameters is wide and covers various immunity-related responses. Our overall results are in agreement with previous heritability calculations [1,3,5,6] and identification of QTLs for various traits including cell counting [9,16], antibody response [17,18] and more recently serum levels of IL10 and IFNγ cytokines after a challenge by the classical swine fever vaccine [10]. It is well known that IR is highly dependent on environment. However, the growing data set on significant heritability levels of many immunity-related parameters together with the detection of QTLs strongly suggest that a large number of immune traits at various positions in the complex scheme of IR is under a consistent genetic control.

### Functional genomics studies

Animals were ranked according to immunity parameter levels and the most extreme high and low animals were selected for transcriptome analysis of blood cells. As mentioned in the Methods section, blood for transcriptome analysis had been sampled together with blood for IR measurements, in order to have a direct correspondence between measured IR traits and blood transcriptome for each animal. A first set of nine parameters was considered for functional genomics studies (Table 1). Among them, seven parameters had significant h^2^ higher than 0.3 on average (WBC, PHAG, IL10-PMAIONO, IL2-PMAIONO, IFNG-PMAIONO, TCRγδ^+^ and CD4^-^ CD8^+^) and two parameters were found either not heritable (TNF) or very weakly heritable ( IgG-Mh). A significant number of genes were found differentially expressed between high and low animal groups for the parameters WBC, PHAG, IL10-PMAIONO, IL2-PMAIONO, and CD4^-^ CD8^+^ and preliminary analyses of the differentially expressed genes show biological relevance. Conversely, no difference between the blood transcriptome of pigs that were extreme for the parameters TNF, IgG-Mh and TCRγδ which were more weakly heritable, was observed. Our results show that differences of some IR parameters levels are associated with differences of transcript levels and that blood transcriptome analysis should be informative to refine some immunity traits.

**Table 1:** Transcriptome analysis of blood for a set of 9 immunity parameters and correspondence with h^2^ estimate

Phenotypes		Number of pigs		Number of differentially expressed genes		
	
	h^2^ [CI95%]	High levels	Low levels	Total	Down-regulated in high group	Up-regulated in high group
WBC	0.7 [0.3-1]	9	9	334	235	99
PHAG	0.6 [0,2 - 0,9]	9	11	336	125	211
IL10-PMAIONO	0.9 [0.9-0.9]	10	10	756	539	217
IL2-PMAIONO	0.7 [0.5-0.8]	10	10	642	312	330
TNF	0 [0-0]	7	7	0	0	0
IFNG-PMAIONO	0.5 [0,1 - 0,8]	7	8	1009	606	403
IgG-Mh	0.1 [0 - 0,2]	10	10	0	0	0
TCRγδ^+^	0.6 [0,4 - 0,7]	10	10	0	0	0
CD4^-^ CD8^+^	0.4 [0 - 0,7]	4	6	173	111	62

## Conclusion and perspectives

To our knowledge, this is one of the first large-scale studies based on a rigorous vaccination protocol, suitable for collecting numerous data on innate and adaptive IR, which combines both genetics and functional genomics methods. Our results agree with previous studies demonstrating that many immune parameters are genetically controlled despite a well-known strong effect of the environment. Our first preliminary results on blood transcriptome suggest that this parameter could provide additional phenotypic information to immunity traits in pig. Since transcriptome analyses look informative for some heritable immunity traits, it might be anticipated that levels of gene expression could be combined with genetic studies to identify candidate genes underlying heritable IR parameters. Genetic analyses are in progress to calculate correlations between immunity parameters and between immunity and production traits. A second pig population has been created in an experimental unit by inseminating sows with the semen of 29 sires of the test farm population. Analysis of this population will provide new data to increase the experimental power, to study genotype x environment interactions and to start a divergent selection experiment on immunity traits. The ultimate goal will be to relate immunocompetence with resistance to disease.

## List of abbreviations used

CD: Cluster of differentiation; CI: Confidence interval; CONA: Concanavaline A; CRP: C reactive protein; Cy: Cyanin; EDTA: Ethylene diamine tetraacetic acid; ELISA: Enzyme-linked immunosorbent assay; EOS: Eosinophils; FACS: Fluorescence-activated cell sorting; HAPT: Haptoglobin; h^2^	Heritability; HT: Hematocrit; IFNα or IFNA: Interferon alpha; IFNG or IFNγInterferon gamma; Ig: Immunoglobulin; IL: Interleukin ; IONO : Ionomycin ; IR : Immune response ; LPS: Lipopolysaccharide; Mh: *Mycoplasma hyopneumoniae;* MHC: Major Histocompatibility Complex; MON : Monocytes ; NEU : Neutrophils ; PHAG : Phagocytosis ; PLT: Platelets; PMA: Phorbol Myristate Acetate; PMAIONO: Mix of PMA and Ionomycin; PROLIF : Lymphocyte proliferation ; QTL: Quantitative Trait Locus; RBC: Red Blood Cells; RDW: Red cell distribution width; TCR: T cell receptor; Th: Helper T cells; TNF: Tumor Necrosis Factor; WBC: White Blood Cells.

## Competing interests

The authors declare that they have no competing interests.

## Authors' contributions

LF participated to the choice of the experimental protocol, to the IR parameter measurements and transcriptome analysis. She carried out the genetic analyses and drafted the manuscript. YG was involved in animal phenotyping and carried out the transcriptome analyses. IPO and FL contributed to the experimental protocol choice and to the IR parameters measurements. MB was in charge of animal maintenance in the test farm. MJM coordinated the contacts with the breeders. JPB participated to the pig family design and coordinated the genetic analyses. CRG coordinated the whole study, contributed to the choice of the experimental protocol, IR parameter measurements, genetic and transcriptome analyses, interpretation of results and corrected the manuscript. All authors have read and approved the final manuscript.

## Supplementary Material

Additional file 1 Table S1 - List of immunity parameters included in the study and associated assays for phenotyping Click here for file
